# Structural basis for a class of nanomolar influenza A neuraminidase inhibitors

**DOI:** 10.1038/srep02871

**Published:** 2013-10-16

**Authors:** Philip S. Kerry, Sankar Mohan, Rupert J. M. Russell, Nicole Bance, Masahiro Niikura, B. Mario Pinto

**Affiliations:** 1 Biomedical Sciences Research Complex, University of St Andrews, St Andrews, Fife, KY16 9ST, UK; 2 Department of Chemistry, Simon Fraser University, Burnaby, British Columbia, V5A 1S6, Canada; 3 Faculty of Health Sciences, Simon Fraser University, Burnaby, British Columbia, V5A 1S6, Canada; 4 Dedication: This paper is dedicated to the memory of Dr. Rupert Russell, who died before this work could be completed.

## Abstract

The influenza virus neuraminidase (NA) is essential for the virus life cycle. The rise of resistance mutations against current antiviral therapies has increased the need for the development of novel inhibitors. Recent efforts have targeted a cavity adjacent to the catalytic site (the 150-cavity) in addition to the primary catalytic subsite in order to increase specificity and reduce the likelihood of resistance. This study details structural and *in vitro* analyses of a class of inhibitors that bind uniquely in both subsites. Crystal structures of three inhibitors show occupation of the 150-cavity in two distinct and novel binding modes. We believe these are the first nanomolar inhibitors of NA to be characterized in this way. Furthermore, we show that one inhibitor, binding within the catalytic site, offers reduced susceptibility to known resistance mutations via increased flexibility of a pendant pentyloxy group and the ability to pivot about a strong hydrogen-bonding network.

Influenza viruses constitute a continuing threat to public health worldwide^1^. In addition to recurring seasonal epidemics, the occasional appearance of pandemic strains serves as a reminder of the importance of plans for influenza prevention and control. Beyond the annual production of vaccines, these plans have included the stockpiling of antiviral drugs, most commonly the neuraminidase inhibitors Oseltamivir (**1**), Zanamivir (**2**) and, more recently, Peramivir (**3**) (Fig. 1). The emergence of resistant strains to these drugs makes the development of novel antivirals an urgent concern.

The Influenza neuraminidase (NA) surface glycoprotein is responsible for the cleavage of sialic acid residues from the surface of the infected cell^2^. Inhibition of this process prevents the release of nascent virions from the surface of the cell, reducing the spread of the infection. There are ten known subtypes of NA (N1-10)^3^, which have been classified by phylogenetic analyses into two distinct groups: group 1 (N1, N4, N5, and N8) and group 2 (N2, N3, N6, N7, and N9)^4^. The N10 subtype is considered to be a “NA-like” protein rather than a true NA as it was found to have only 20–27% sequence identity with other NA subtypes. In addition, recombinant N10 protein showed no or extremely low sialidase activity in assay using 2′-(4-methylumbelliferyl)-α-D-*N*-acetylneuraminic acid (4-MU-NANA) as a substrate or with natural substrates in a glycan microarray assay^5^,^6^. Early structures of the catalytic head domain from group 2 subtypes N9 and N2 were the basis of drug development programmes leading to the production of Oseltamivir and Zanamivir. However, more recently the elucidation of several structures of group 1 NAs has highlighted key group-specific differences in the shape of the NA active site^4^. Specifically, in the active sites of group 1 NAs a loop, consisting of residues 147–152 (the 150-loop), adopts a conformation leading to the opening of a pocket to the side of the active site (also known as the 150 cavity). While this cavity was present in structures of the *apo* form of most group-1 NAs (some controversy surrounds the structure of the NA from a H1N1 2009 pandemic strain^7^,^8^,^9^,^10^), it appears to be closed by movement of the 150-loop in response to ligand binding^4^. In the subsequent complex the 150-loop occupies a position similar to that observed in structures of group-2 NAs.

The discovery of the 150-cavity has lead to the development of several inhibitors designed to exploit contacts in this region and increase specificity^11^,^12^,^13^,^14^. In particular, *p*-tolyl-Neu5Ac2en (Fig. 1), an inhibitor based on the transition state analogue Neu5Ac2en, was demonstrated to insert into the 150-cavity and prevent closure of the 150-loop^12^. As a consequence, inhibition of NA activity by this compound was described as group-specific, with substantially greater inhibition of group-1 than group-2 NAs. However, recently several studies have indicated that movement of the 150-loop may not be restricted to group-1 NA. More specifically, molecular dynamics studies have indicated that all NAs may retain the propensity for both “open” and “closed” states, maintaining an equilibrium that varies between strains^8^,^15^,^16^. These *in silico* studies have been supported by evidence of a partially open 150-loop in a complex of N2 and Oseltamivir^17^.

In previous studies, we have described the synthesis and biological characterization of a novel class of NA inhibitors related to Oseltamivir^13^,^18^, **4–8** (Fig. 1). In these studies, this class was demonstrated to inhibit NA activity at nanomolar concentrations in a highly selective manner, with compound **7** showing the propensity for significant group-specificity. Furthermore these compounds did not inhibit the activity of mammalian neuraminidases, NEU3 and NEU4^19^, an off-target effect that has been observed with Zanamivir^20^. The parental compound within this class is the Oseltamivir isomer **4**, in which the cyclohexene double bond has been moved to the C2–C3 position. Further extensions to this scaffold were employed at the C4 position, using either guanidino (**5**) or triazole (**6–8**) groups, the latter of which were expected to project into the 150-cavity. Here we report further biological characterization of **4** and **5**, indicating the guanidine derivative **5** offers reduced susceptibility to the known Oseltamivir-resistance mutation H274Y. Furthermore we reveal structures of **4–8** in complex with a group-1 NA. While complexes of N8:**4** and N8:**5** interact in a manner similar to previous NA inhibitors, the structures of N8:**6–8** indicate novel binding modes employing contacts with residues within the 150-loop and cavity. These structures, in combination with our recently published molecular dynamics studies using the same compounds^15^, suggest that the movements within this region may be more complicated than previously thought.

## Results

### Inhibition of Oseltamivir-resistant viruses by compounds 4 and 5

To investigate the efficiency of the novel NA antagonists **4** and **5** against Oseltamivir resistant strains, both compounds were tested in an *in vitro* replication inhibition assay using A/Brisbane/59/2007 (Oseltamivir-sensitive, WT) and A/Brisbane/59/2007-like Oseltamivir-resistant (H274Y) strains. Compounds **4** and **5** inhibited the replication of WT with ED_50 _values similar to, or lower than that achieved by Oseltamivir (**1**) (Table 1, Supplementary Fig. S3). In contrast to **1** and **4**, compound **5** showed inhibition of the resistant strain.

### Structures of N8 in complex with Oseltamivir-related compounds 4 and 5

**Figure 2:**
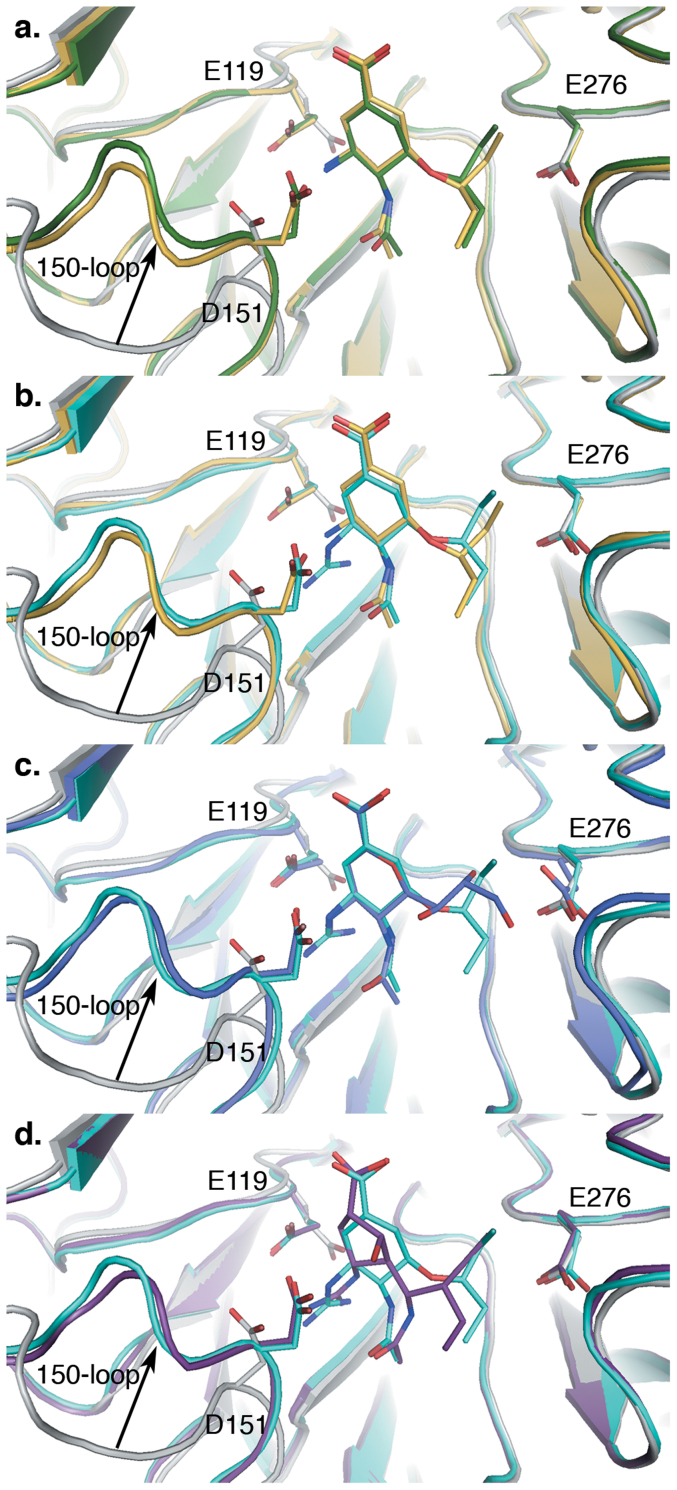
Comparison of the binding of 4 and 5 to N8 with structures of existing NA inhibitors. Superposition of N8:**4** (dark green, (a)) or N8:**5** (cyan, (b–d)) with *apo*-N8 (2HT5, white, all panels), N8:Oseltamivir(**1**) (2HT8, gold, (a) and (b)), N8:Zanamivir(**2**) (2HTQ, dark blue, (c)) and N8:Peramivir(**3**)(2HTU, purple, (d)). Inhibitor binding leads to repositioning of active site residues E119, D151, and E276 (shown as sticks) and closure of the 150-loop (indicated by an arrow).

To understand the molecular basis behind the inhibitory activity of this novel class of inhibitors, crystal structures of the parental compounds **4** and **5** were obtained in complex with a group-1 NA by soaking crystals of N8 [A/duck/Ukraine/1/63 (H3N8)] enzyme in 1 mM solutions of compound (Fig. 2). In both N8:**4** and N8:**5** complexes, the inhibitors bound in a position and orientation very similar to that observed previously in structures of N8:**1** (Fig. 2a and b). In particular, N8:**4** overlays almost exactly with N8:**1**, with only a subtle repositioning of the C7 atom due to the change in position of the double bond in the cyclohexene ring relative to **1**. Surprisingly, given the lower activity of compound **4** in *in vitro* inhibition assays^13^, no further significant differences in the position of the compound's pendant groups or active site residues were observed. The position of **5** in the N8 active site is similarly conserved, with the addition of a guanidino group extending towards the base of the active site in a position reminiscent of the guanidino group of Zanamivir (N8:**2**) (Fig. 2c), but distinct from that of Peramivir (N8:**3**) (Fig. 2d). The additional contacts made between this group and the active site (specifically E227 and the backbone carbonyls of W178 and D151) explains the increase in inhibitory activity compared to **4**.

### Structures of N8 in complex with compounds (6–8) extending into the 150 cavity

**Figure 3:**
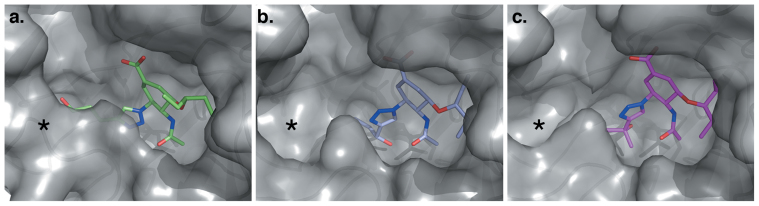
Two distinct binding modes of triazole-containing influenza inhibitors 6-8. Surface representations of the enzyme active site in N8:**6** (a), N8:**7** (b) and N8:**8** (c) complexes, with ligand in stick format. The 150-cavity (indicated by an asterisk (*)) of N8:**7** and N8:**8** complexes remains open, in contrast to the complex with **6**, where the 150-loop is closed.

**Figure 4:**
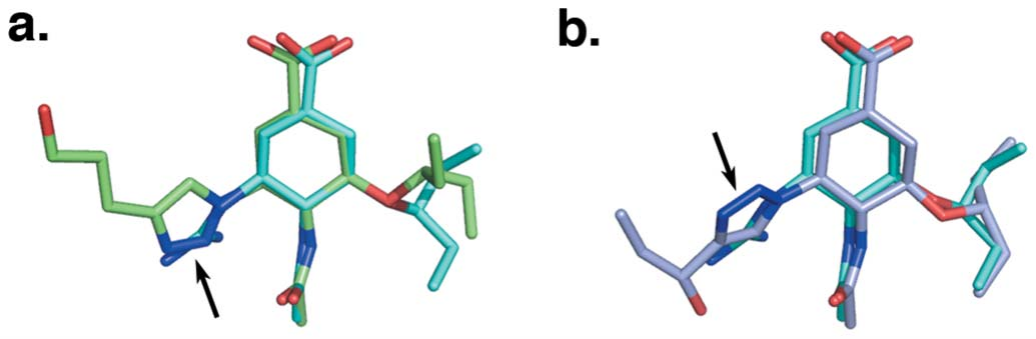
Orientation of the 1,2,3-triazole groups of 6 and 7. Superimposition of (a) **5** (cyan) and **6** (green) and (b) **5** and **7** (pale blue) as bound within the N8:**5**, N8:**6**, and N8:**7** complexes, respectively. The triazole nitrogen atoms (indicated by an arrow) of **6** overlay with the guanidino moiety of **5**, whereas in N8:**7** this group is rotated approximately 135°, leading to a repositioning of the alkyl-alcohol functional group.

**Figure 5:**
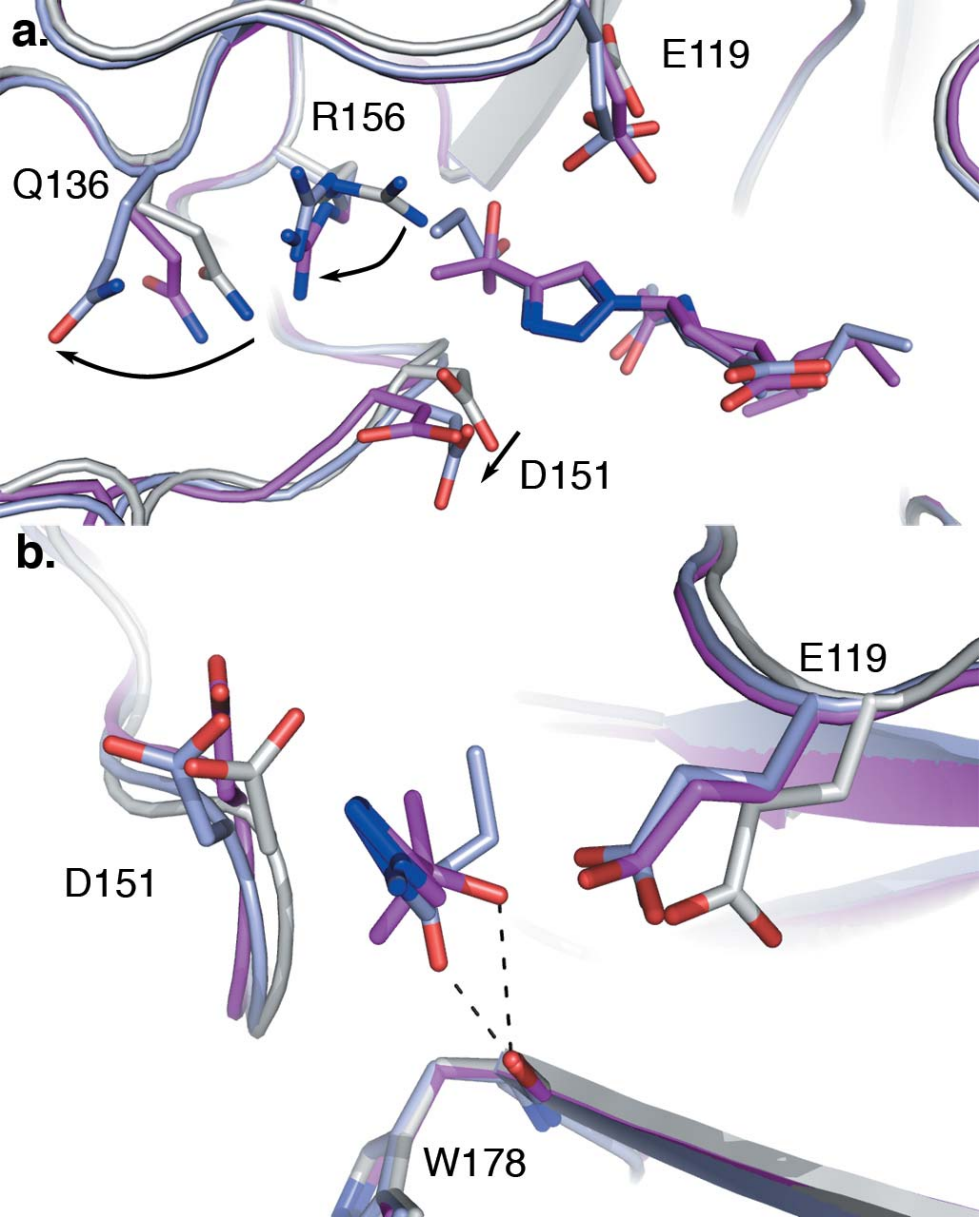
Interactions of 7 and 8 with the 150-cavity of the N8 active site. Superimposition of N8:**7** (pale blue) and N8:**8** (magenta) with *apo*-N8 (white, 2HT5), with the atoms of ligands and relevant side chains shown as sticks. (a) Rearrangements within the 150-cavity of N8:**7** and N8:**8** in response to ligand binding (side chain movements indicated with arrows). Insertion of the triazole-hydroxyl moiety into the 150-cavity results in a repositioning of the guanidino group of R156, and resultant displacement of the Q136 side chain. Additionally the carboxylic acid of D151 moves 1.5 Å out of the active site to accommodate the triazole group. (b) View from the enzyme active site towards the 150-cavity, showing the interactions between the ligand triazole-hydroxyl moieties and residues within the active site (both shown as sticks, the cyclohexene ring and associated groups have been removed to improve visibility). Hydrogen bonding interactions formed between the triazole-hydroxyl moieties of **7** and **8**, and W178 main-chain carbonyl are indicated with dashed lines.

In addition to compounds **4** and **5**, we also examined the binding mode of three further members of this class of inhibitor, **6**, **7**, and **8,** in complex with N8 by X-ray crystallography (Fig. 3). As with structures of N8:**4** and N8:**5**, the structure of N8:**6** bears much similarity with N8:**1**, however the pentyloxy pendant group of **6** appears to have re-oriented out of the hydrophobic pocket (Fig. 3a). The three nitrogens within the triazole group of N8:**6** overlay with the position of the guanidino group of N8:**5** (Fig. 4), allowing closure of the 150-loop. In contrast, in N8:**7** and N8:**8** the presence of branched alcohol groups beyond the triazole ring causes reorientation of the ring structure and prevents closure of the 150-loop (Fig. 3b and c). Examination of the residues within the 150-cavities of N8:**7** and N8:**8** reveals that the rearrangement of several side chains is required to accommodate the triazole-hydroxyl moieties (Fig. 5a) and allow the formation of further hydrogen bonding interactions (Fig. 5b). For each complex similar structures were obtained at a variety of soaking times (data not shown).

## Discussion

In our previous studies we demonstrated the synthesis and *in vitro* properties of a new class of inhibitors of influenza A NA. While similar to the established NA inhibitor Oseltamivir carboxylate (**1**), these inhibitors contained significant alterations such as the change in position of the double bond within the carbocycle and incorporation of a guanidino or substituted-triazole functions at the C-4 position (Oseltamivir numbering), leading to increased specificity for group-1 NA in particular for the triazole-substituted inhibitors^13^. Previously some of these inhibitors have been shown to inhibit growth of laboratory N1 and N2 strains of influenza A at micromolar concentrations^18^. Biological characterization of the parental compounds of this class (**4** and **5**) presented here indicate that both inhibit growth of an H1N1 isolate (A/Brisbane59/2007) and compared favourably to the same assays performed using **1**, with either similar (in the case of **4**) or approximately 3-fold lower (in the case of **5**) ED_50 _values. However, in contrast to **1** and **4**, inhibitor **5** showed activity against an Oseltamivir-resistant virus with the H274Y mutation. While the inhibition of **4** against the H274Y virus was poor (ED_50_ > 500 μM), the concentration of **5** required was approximately 10-fold higher than for wild-type virus (ED_50_ = 34 μM compared to ED_50_ = 3.9 μM). While the ED_50_ value of **5** was increased by the incorporation of the H274Y mutation, alternative compounds such as **3** were affected to a greater (100-fold) extent^21^. Therefore **5** represents a real and substantial improvement on the resistance profile of similar compounds.

The structures of the N8:**4** and N8:**5** complexes are remarkably similar to N8:**1**, suggesting that the reduced susceptibility of these compounds is not due to substantial changes to the position of the compounds within the active site. Compounds within this class contain a cyclohexene ring with a C2–C3 carbon-carbon double bond, in contrast to the C2–C7 position present in **1**. Although the change in position of the double bond does not appear to influence the final position of the pentyloxy side chain in N8:**4** or N8:**5**, the position of this group within N8:**6** indicates that repositioning of the double bond between C2–C3 in the carbocycle may increase the flexibility in this region, in agreement with the results of our previous molecular dynamics simulations^15^. The complex of **1** and an N1 bearing the H274Y mutation shows that E276 is forced into the hydrophobic pocket and the pentyloxy group shifts 2 Å out of the enzyme active site^22^. Rather than a change in the rotameric conformation of this pendant group, this is achieved by slight twisting of the entire ligand within the active site, removing other groups from their optimal positions. It is possible that the differences in the position of the double bond between **1** and **5** allow the pentyloxy group to move without the same distortions, reducing the impact of the mutation on ligand binding. Indeed, while most of the atoms within the cyclohexene ring of N8:**5** overlay closely with their counterparts in N8:**1**, the position of C7 is more similar to that observed in the structure of **1** bound to an N1 bearing the H274Y mutation (Supplementary Fig. S1).

**Figure 6:**
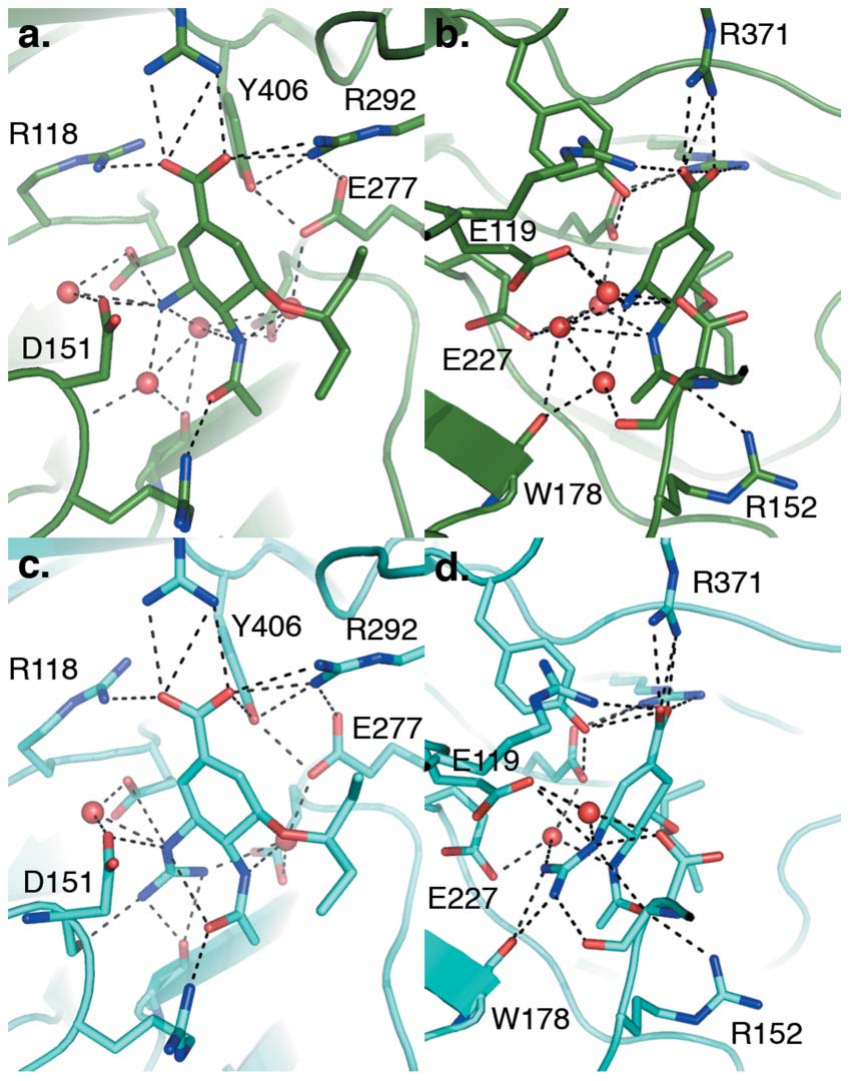
Active-site interactions of 4 and 5 in the N8 complex showing the H-bonding networks at the 4-amino and 4-guanidinium function and the interactions of ariginine triads with the carboxylate function. Representations of N8:**4** (a and b) and N8:**5** (c and d), with ligands and interacting residues shown as sticks, interacting water molecules shown as spheres and H-bond interactions indicated with dashed lines. Panels (a) and (b) and (c) and (d) are related by a 90° rotation in the y –axis.

The increased activity of **5** against both wild-type and Oseltamivir-resistant influenza in comparison to **4** is likely to be due to the additional contacts made by the guanidino group present within **5** (Fig. 6). The position of this group within the NA active site is very similar to its position in the structure of the N8:**2** complex (but distinct from the same group in N8:**3**). As with N8:**2**, the guanidino group of N8:**5** forms additional hydrogen bonding contacts with the carboxyls of E119 and E227 and the backbone carbonyls of W178 and D151. Comparison with N8:**4** indicates that these direct hydrogen bonds replace a looser network of water-mediated interactions, with retention of only the direct contact between the amino group of **4** and E119. Perhaps surprisingly, these additional contacts only result in 3-fold decreases in *K*_i_^13^ and ED_50_, in comparison to the 100–200-fold decreases observed between **2** and its amino derivative, 4-amino-DANA^23^. This may indicate that the increased enthalpic benefit of the additional hydrogen bonding interactions may come at an entropic cost not observed in their more hydrophilic DANA-based counterparts. As outlined above, the scaffold employed in these compounds may allow greater flexibility of the pentyloxy side chain. Furthermore, unlike the weaker interactions surrounding the amino group of **4,** the arrangement of the hydrogen-bonding network seems to anchor **5** by the guanidino and carboxylic acid groups, allowing the ligand to pivot from this position. Therefore the combination of this scaffold and the added interactions formed by the guanidino moiety may be to compensate for the H274Y resistance mutation at the cost of decreased potency against wild-type NA. Interestingly, the guanidine derivative of **1** showed little or no improvement compared to **1** in functional assays using NA (either wild-type or H274Y)^24^. This would imply that it is not just guanidino function, but the combination of both the new scaffold (change in position of the double bond) and the guanidino group, contributing towards the observed undiminished activity of compound **5** against the H274Y mutation. Overall, the ligand may be capable of binding more tightly, and in more positions, than **1**.

Recent studies have highlighted the 150-cavity of group-1 NAs as a potential source for increased specificity in NA antagonists. Previously, several compounds have been developed to occupy this cavity using bulky hydrophobic groups to lock the 150-loop in an open conformation^11^,^12^. Seeking to similarly exploit this cavity by an alternative approach, we have developed a series of inhibitors incorporating a triazole group at the C4 position of **4**. Interestingly, complexes of three representatives of this class displayed two distinct binding modes (Figs. 3 and 4). Unexpectedly, the structure of the N8:**6** complex indicated that the 150-loop was able to close around the triazole group, sandwiching it between D151 and E119. In this position, the nitrogen atoms of the triazole group occupy similar positions to the guanidino group of **5**, retaining contact with the carbonyl of W178. In contrast, the triazole groups of N8:**7** and N8:**8** are rotated by approximately 135°, resulting in the branched alcohol groups extending into the 150-cavity. Consequentially, in order to accommodate these groups, the positions of several residues within the pocket are relocated. In particular, to avoid a steric clash with the isopropanol group of **7**, the guanidino group of R156 has rotated 120° away from the ligand (Fig. 5a). To enable this movement the side chain of Q136 has moved approximately 2 Å away from the ligand, relative to its position in N8:**5**. Similar movements are observed in N8:**8** to accommodate the *tert*-propanol group. Additionally, in both N8:**7** and N8:**8**, D151 has relocated slightly further out of the active site to allow engagement with the 3-nitrogen of the triazole group. Whilst the electron density surrounding the alkyl-alcohol groups within these structures only allows for tentative placement of the hydroxyl moieties, in both N8:**7** and N8:**8** this group is likely to form at least one hydrogen bond with the enzyme active site (Fig. 5b, Supplementary Fig. S2).

In summary, **5** has a reduced resistance susceptibility for the H274Y mutation in comparison to the established NA antagonist Oseltamivir. This is achieved by a combination of the repositioning of the double-bond within the carbocyle, giving greater flexibility, and additional contacts formed by the guanidinium function. Furthermore, **5** is bound very tightly by two anchor points: the H-bonding networks at the guanidinium function and the interactions of arginine triads with the carboxylate function. There is a pivoting of the ring about these anchor points. We propose that this compound is held so tightly that it is able to overcome the H274Y mutation just by maintaining an alternative pose.

Regarding the triazole-extended derivatives **6–8**, the 150 cavity is certainly occupied but one sacrifices binding activity because the hydrogen-bonding network seen with the guanidinium or amino function in **4** or **5** has been compromised. The results are corroborated by the MD studies in which this group is not stable in the 150 cavity but exits the subsite periodically^15^.

## Methods

### Viruses

An influenza A virus type strain A/Brisbane/59/2007 (H1N1) was provided by the WHO National Influenza Centre, National Microbiology Laboratory (NML), Winnipeg, Canada. A/Brisbane/59/2007-like oseltamivir-resistant strain was isolated and provided by the Provincial Health Services Authority (PHSA)-British Columbia Centre for Disease Control (BCCDC) Virology Laboratory, Vancouver, Canada. The Oseltamivir-resistant isolate was identified as A/Brisbane/59/2007-like by hemagglutination inhibition assays using strain-specific antisera and by genome sequencing. The Oseltamivir resistance was determined by an enzyme inhibition assay by NML. The H274Y mutation was confirmed by sequencing as well as by the BCCDC H274Y SNP assay.

### Virus inhibition assay

The inhibitory effect was tested in MDCK cells as previously described^18^. Monolayers were infected with 50 TCID_50_ of either WT or H274Y virus in the presence of various concentrations of compounds in quadruplicates in 96 well plates. After 4 days incubation the effect was quantitatively evaluated by a neutral red (NR) dye uptake assay^25^,^26^. Briefly, the monolayers were stained with 0.01% NR for 2 hours in DMEM and washed twice with phosphate buffered saline. After air-drying, the incorporated NR was extracted with 100 μl of guanidine-HCl (3 M in water) and the concentration of released NR was measured by a microplate reader at OD_490_. ED_50_ was calculated based on the measurement of uninfected control monolayers as 100% protection (OD_490_ = 0.847) and no test compound control (with 50TCID_50_ A/Brisbane) as 0% protection (OD_490_ = 0.086 and 0.082 for WT and H274Y, respectively).

### Protein X-ray crystallography

N8 sialidase from A/Duck/Ukraine/1/63 (H3N8) was prepared from virus grown in hen's eggs. Sialidase was released from purified virions by bromelain digestion, and purified as described previously^4^. Crystals of N8 protein were grown by vapour diffusion in hanging drops consisting of 1 μl concentrated protein solution (8 mgml^−1^ in 50 mM Tris-HCl pH8.0) and 1 μl reservoir solution (40% (±)-2-methyl-2,4,-pentanediol (MPD)). Crystals of N8 sialidase were soaked reservoir solution containing 1 mM compound for 5 min (**4** and **7**), 60 min (**6** and **8**) or 16 hours (**5**), although similar results were also obtained at alternative soaking times. Data were collected on an in-house rotating anode (RA Micro7 HFM) and a Saturn944 CCD at 100 K and processed with HKL2000^27^. Refinement was carried out using PHENIX^28^, combined with manual model building with Coot^29^. Statistical support for the structures obtained is presented in Supplementary Table S1. Structural data have been deposited with the Protein Data Bank with accession codes 4KS1 (N8:**4**), 4KS2 (N8:**5**), 4KS3 (N8:**6**), 4KS4 (N8:**7**), 4KS5 (N8:**8**).

## Author Contributions

B.M.P. and S.M. conceived and designed the project. M.N. and N.B. designed and performed virology experiments. P.S.K. and R.J.M.R. designed and performed the X-ray crystallography described. P.S.K., R.J.M.R., S.M., and B.M.P. analysed the results. P.S.K., S.M., and B.M.P. contributed to the preparation of the manuscript.

## Supplementary Material

Supplementary Information Supplementary Information

## Figures and Tables

**Figure 1:**
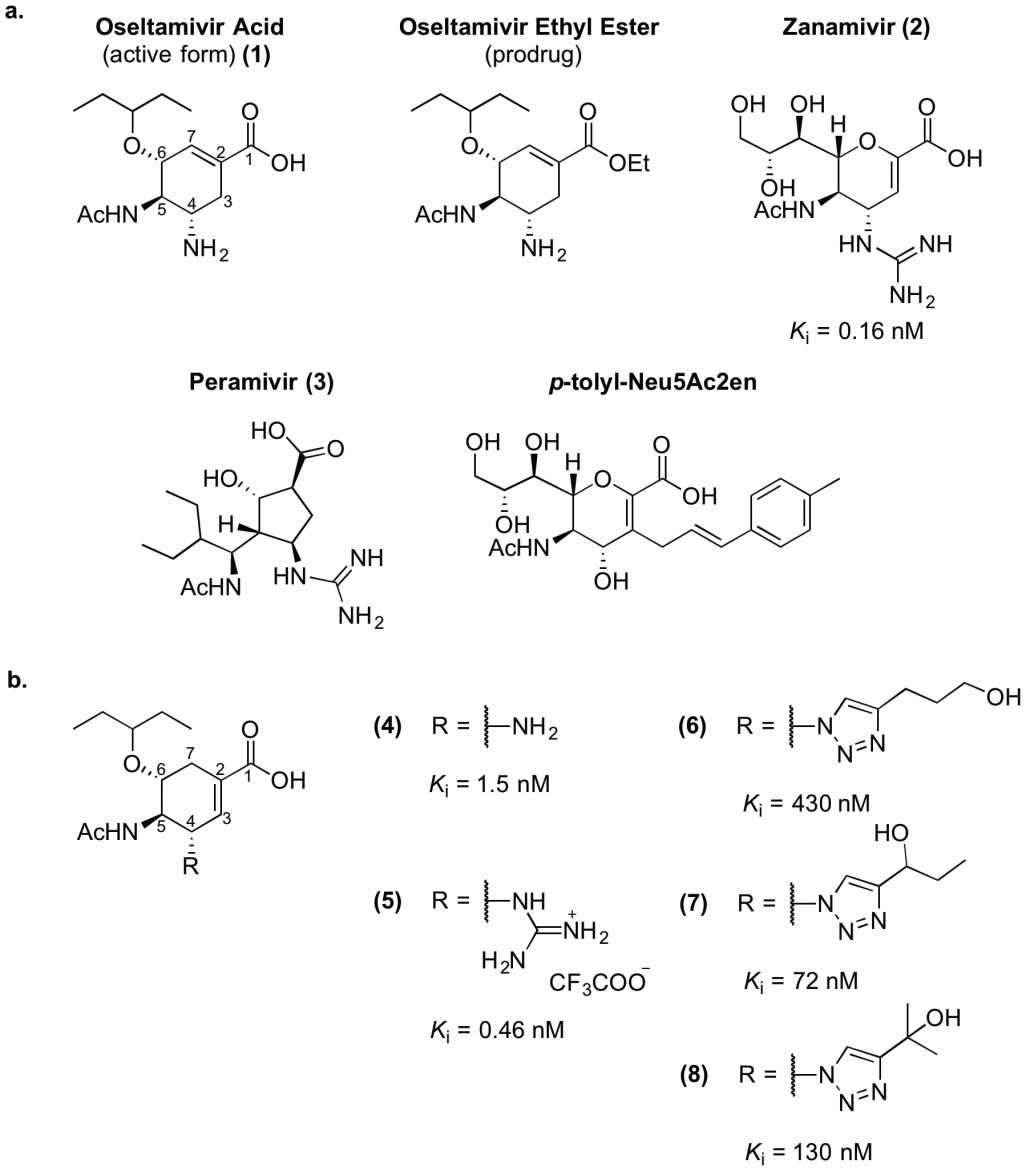
Chemical structures of anti-influenza drugs and novel inhibitors. Structures of previously characterised compounds (a) and novel inhibitors characterised in this study (b). *K*_i_ values were measured with virus-like particles containing N1 subtype neuraminidase^13^.

**Table 1:** Comparison of ED_50_ (μM) to wild-type and Oseltamivir-resistant (H274Y) strains

Influenza strain\Compounds	1	4	5
A/Brisbane/59/2007 (WT)	12	12	3.9
A/Brisbane/59/2007-like (H274Y)	>500	>500	34

## References

[b1] Influenza fact sheet No. 211: World Health Organization; (2009).

[b2] Air G. M. Influenza neuraminidase. Influenza Other Respi Viruses. 6(4), 245–256 (2012). 22085243 10.1111/j.1750-2659.2011.00304.x PMC3290697

[b3] Tong S. *et al.* A distinct lineage of influenza A virus from bats. Proc Natl Acad Sci U S A. 109(11), 4269–4274 (2012). 22371588 10.1073/pnas.1116200109 PMC3306675

[b4] Russell R. J. *et al.* The structure of H5N1 avian influenza neuraminidase suggests new opportunities for drug design. Nature. 443(7107), 45–49 (2006). 16915235 10.1038/nature05114

[b5] Zhu X. *et al.* Crystal structures of two subtype N10 neuraminidase-like proteins from bat influenza A viruses reveal a diverged putative active site. Proc Natl Acad Sci U S A. 109(46), 18903–18908 (2012). 23012478 10.1073/pnas.1212579109 PMC3503178

[b6] Li Q. *et al.* Structural and functional characterization of neuraminidase-like molecule N10 derived from bat influenza A virus. Proc Natl Acad Sci U S A. 109(46), 18897–18902 (2012). 23012237 10.1073/pnas.1211037109 PMC3503196

[b7] van der Vries E. *et al.* H1N1 2009 pandemic influenza virus: resistance of the I223R neuraminidase mutant explained by kinetic and structural analysis. PLoS Pathog. 8(9), e1002914 (2012). 23028314 10.1371/journal.ppat.1002914 PMC3447749

[b8] Amaro R. E. *et al.* Mechanism of 150-cavity formation in influenza neuraminidase. Nat Commun. 2, 388 (2011). 21750542 10.1038/ncomms1390 PMC3144582

[b9] Li Q. *et al.* The 2009 pandemic H1N1 neuraminidase N1 lacks the 150-cavity in its active site. Nat Struct Mol Biol. 17(10), 1266–1268 (2010). 20852645 10.1038/nsmb.1909

[b10] Han N. & Mu Y. Plasticity of 150-loop in influenza neuraminidase explored by hamiltonian replica exchange molecular dynamics simulations. PLoS One. 8(4), e60995 (2013). 23593372 10.1371/journal.pone.0060995 PMC3622661

[b11] Rudrawar S. *et al.* Synthesis and evaluation of novel 3-C-alkylated-Neu5Ac2en derivatives as probes of influenza virus sialidase 150-loop flexibility. Org Biomol Chem. 10(43), 8628–8639 (2012). 22976385 10.1039/c2ob25627d

[b12] Rudrawar S. *et al.* Novel sialic acid derivatives lock open the 150-loop of an influenza A virus group-1 sialidase. Nat Commun. 1, 113 (2010). 21081911 10.1038/ncomms1114 PMC3060605

[b13] Mohan S., McAtamney S., Haselhorst T., von Itzstein M. & Pinto B. M. Carbocycles related to oseltamivir as influenza virus group-1-specific neuraminidase inhibitors. Binding to N1 enzymes in the context of virus-like particles. J Med Chem. 53(20), 7377–7391 (2010). 20873795 10.1021/jm100822f

[b14] Wen W. H. *et al.* Analogs of zanamivir with modified C4-substituents as the inhibitors against the group-1 neuraminidases of influenza viruses. Bioorg Med Chem. 18(11), 4074–4084 (2010). 20452227 10.1016/j.bmc.2010.04.010

[b15] Greenway K. T., LeGresley E. B. & Pinto B. M. The influence of 150-cavity binders on the dynamics of influenza A neuraminidases as revealed by molecular dynamics simulations and combined clustering. PLoS One. 8(3), e59873 (2013). 23544106 10.1371/journal.pone.0059873 PMC3609799

[b16] Amaro R. E., Cheng X., Ivanov I., Xu D. & McCammon J. A. Characterizing loop dynamics and ligand recognition in human- and avian-type influenza neuraminidases via generalized born molecular dynamics and end-point free energy calculations. J Am Chem Soc. 131(13), 4702–4709 (2009). 19296611 10.1021/ja8085643 PMC2665887

[b17] Wu Y. *et al.* Induced opening of influenza virus neuraminidase N2 150-loop suggests an important role in inhibitor binding. Sci Rep. 3, 1551 (2013). 23531861 10.1038/srep01551 PMC3609017

[b18] Niikura M., Bance N., Mohan S. & Pinto B. M. Replication inhibition activity of carbocycles related to oseltamivir on influenza A virus in vitro. Antiviral Res. 90(3), 160–163 (2011). 21443905 10.1016/j.antiviral.2011.03.180

[b19] Albohy A., Mohan S., Zheng R. B., Pinto B. M. & Cairo C. W. Inhibitor selectivity of a new class of oseltamivir analogs against viral neuraminidase over human neuraminidase enzymes. Bioorg Med Chem. 19(9), 2817–2822 (2011). 21489803 10.1016/j.bmc.2011.03.039

[b20] Hata K. *et al.* Limited inhibitory effects of oseltamivir and zanamivir on human sialidases. Antimicrob Agents Chemother. 52(10), 3484–3491 (2008). 18694948 10.1128/AAC.00344-08 PMC2565904

[b21] Gubareva L. V., Webster R. G. & Hayden F. G. Comparison of the activities of zanamivir, oseltamivir, and RWJ-270201 against clinical isolates of influenza virus and neuraminidase inhibitor-resistant variants. Antimicrob Agents Chemother. 45(12), 3403–3408 (2001). 11709315 10.1128/AAC.45.12.3403-3408.2001 PMC90844

[b22] Collins P. J. *et al.* Crystal structures of oseltamivir-resistant influenza virus neuraminidase mutants. Nature. 453(7199), 1258–1261 (2008). 18480754 10.1038/nature06956

[b23] von Itzstein M. *et al.* Rational design of potent sialidase-based inhibitors of influenza virus replication. Nature. 363(6428), 418–423 (1993). 8502295 10.1038/363418a0

[b24] Shie J. J. *et al.* Synthesis of tamiflu and its phosphonate congeners possessing potent anti-influenza activity. J Am Chem Soc. 129(39), 11892–11893 (2007). 17850083 10.1021/ja073992i

[b25] Smee D. F., Morrison A. C., Barnard D. L. & Sidwell R. W. Comparison of colorimetric, fluorometric, and visual methods for determining anti-influenza (H1N1 and H3N2) virus activities and toxicities of compounds. J Virol Methods. 106(1), 71–79 (2002). 12367731 10.1016/s0166-0934(02)00137-4

[b26] Smee D. F., Huffman J. H., Morrison A. C., Barnard D. L. & Sidwell R. W. Cyclopentane neuraminidase inhibitors with potent in vitro anti-influenza virus activities. Antimicrob Agents Chemother. 45(3), 743–748 (2001). 11181354 10.1128/AAC.45.3.743-748.2001 PMC90367

[b27] Otwinowski Z. & Minor W. In: Sawyer L., Isaacs N., Bailey S. (eds). Data Collection and Processing. 556–562 (SERC Daresbury Laboratory: Warrington, 1993).

[b28] Adams P. D. *et al.* PHENIX: a comprehensive Python-based system for macromolecular structure solution. Acta Crystallogr D Biol Crystallogr. 66(Pt 2), 213–221 (2010). 20124702 10.1107/S0907444909052925 PMC2815670

[b29] Emsley P., Lohkamp B., Scott W. G. & Cowtan K. Features and development of Coot. Acta Crystallogr D Biol Crystallogr. 66(Pt 4), 486–501 (2010). 20383002 10.1107/S0907444910007493 PMC2852313

