# Supplementary material for: Structural basis for a class of nanomolar influenza A neuraminidase inhibitors
Source: Sci Rep. 2013 Oct 16;3:2871. doi: 10.1038/srep02871 (PMC3797432; doi:10.1038/srep02871)

Structural basis for a class of nanomolar influenza A neuraminidase inhibitors

(Supplementary Information)

Philip S. Kerry[1*], Sankar Mohan[2], Rupert J. M. Russell[1], Nicole Bance[3], Masahiro Niikura[3], B. Mario Pinto[2*]

[1]Biomedical Sciences Research Complex, University of St Andrews, St Andrews, Fife, KY16 9ST, UK, [2]Department of Chemistry, Simon Fraser University, Burnaby, British Columbia, V5A 1S6, Canada and [3]Faculty of Health Sciences, Simon Fraser University, Burnaby, British Columbia, V5A 1S6, Canada.

*email: psk5@st-and.ac.uk, bpinto@sfu.ca

Supplementary Figure Legends

Supplementary Table S1: Data collection and refinement statistics (molecular replacement)

Supplementary Figure S1. Comparison of N8:5 and Oseltamivir in complex with an N1 (H274Y mutant). Superimpositions of N8:**5** (dark green, **a** and **b**), *apo*-N8 (2HT5, white, **a** and **b**), N8:**1** (2HT8, gold, **b** only) and a complex of Oseltamivir and N1 (H274Y mutant) (3CL0, teal, **a** and **b**).

Supplementary Figure S2. Stereo illustrations of novel NA inhibitors within the NA active site. (**a**) N8:**4**, (**b**) N8:**5**, (**c**) N8:**6**, (**d**) N8:**7**, (**e**) N8:**8**. 2Fo-Fc electron density around the ligands contoured at 1σ.

Supplementary Figure S3. ED_{50} of compound 5 and Oseltamivir carboxylate for wild-type and H274Y A/Brisbane/2007. Neutral red staining of the monolayers was quantitated at OD_{490} after 4 days culture with 50 $TCID_{50}$ IFV-A and the test compounds (M). Solid lines indicate the results with the resistant H274Y strain and broken lines with the sensitive wild-type strain. Black lines are for compound **5**and grey lines are for oseltamivir carboxylate. OD_{490} level that indicates 50 % protection is also shown.

Supplementary Table S1

Protein	N8:**4**	N8:**5**	N8:**6**	N8:**7**	N8:**8**
PDB accession code	4KS1	4KS2	4KS3	4KS4	4KS5
Space group	I4	I4	I4	I4	I4
Unit-cell parameters (Å)	a=90.3, b=90.3, c=94.1	a=89.7, b=89.7, c=93.7	a=89.7, b=89.7, c=94.9	a=90.7, b=90.7, c=108.0	a=90.3, b=90.3, c=94.7
Maximum resolution (Å)*	2.20 (2.24-2.20)	2.60 (2.64-2.60)	2.60 (2.64-2.60)	2.50 (2.54-2.50)	2.70 (2.74-2.70)
Unique reflections	17922	10242	11650	15146	10514
Completeness (%) *	93.5 (60.4)	88.8 (90.1)	100.0 (100.0)	99.8 (96.9)	100.0 (100.0)
Mean I/σI *	16.4 (3.0)	19.1 (5.0)	23.4 (4.2)	26.7 (3.5)	19.0 (5.3)
Redundancy	4.0	3.8	4.6	7.1	6.7
R_{sym} (%)*	0.131 (0.359)	0.134 (0.478	0.100 (0.499)	0.113 (0.572)	0.185 (0.574)
Refinement					
Protein atoms	3018	3018	3009	3018	3009
Water atoms	281	197	101	116	63
Ligand atoms	20	23	28	28	28
Other atoms	16	1	1	1	1
Resolution Range (Å)	22.07-2.20	23.44-2.60	32.60-2.6	37.97-2.50	38.03-2.70
R_{work} (%)	15.3	16.4	13.6	17.9	16.8
R_{free} (%)	19.9	19.3	17.6	24.7	19.7
R.m.s.d. bond lengths (Å)	0.009	0.024	0.019	0.014	0.017
R.m.s.d. bond angles (^{o})	1.121	1.960	1.717	1.639	1.416

Supplementary Figure S1

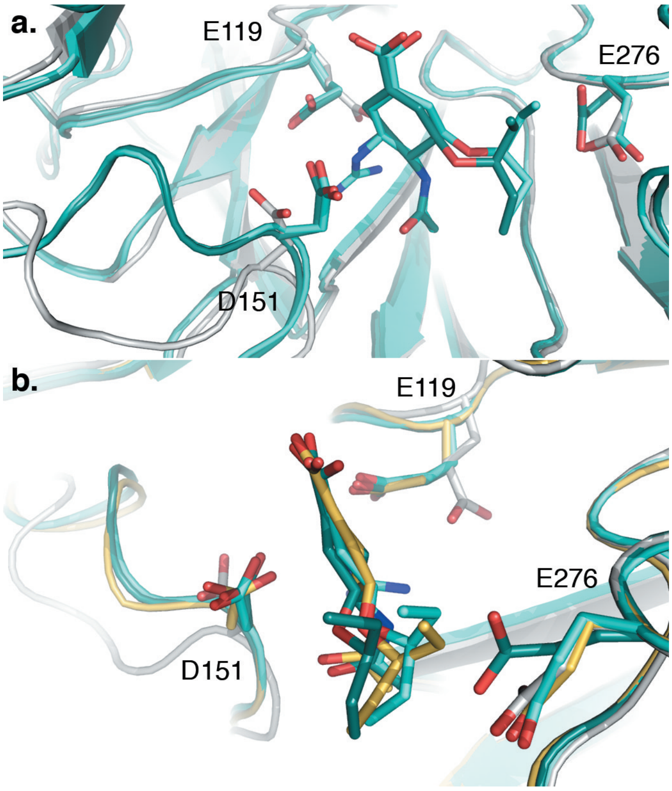

Supplementary Figure S2

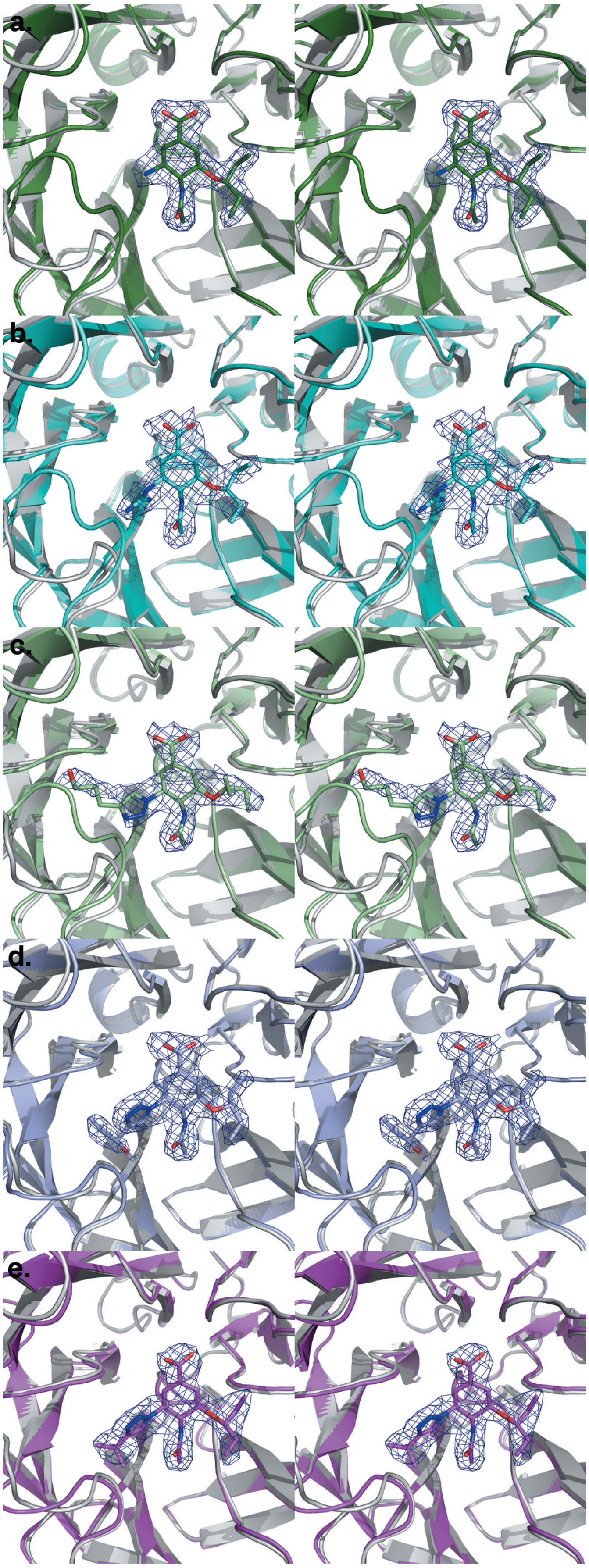

Supplementary Figure S3

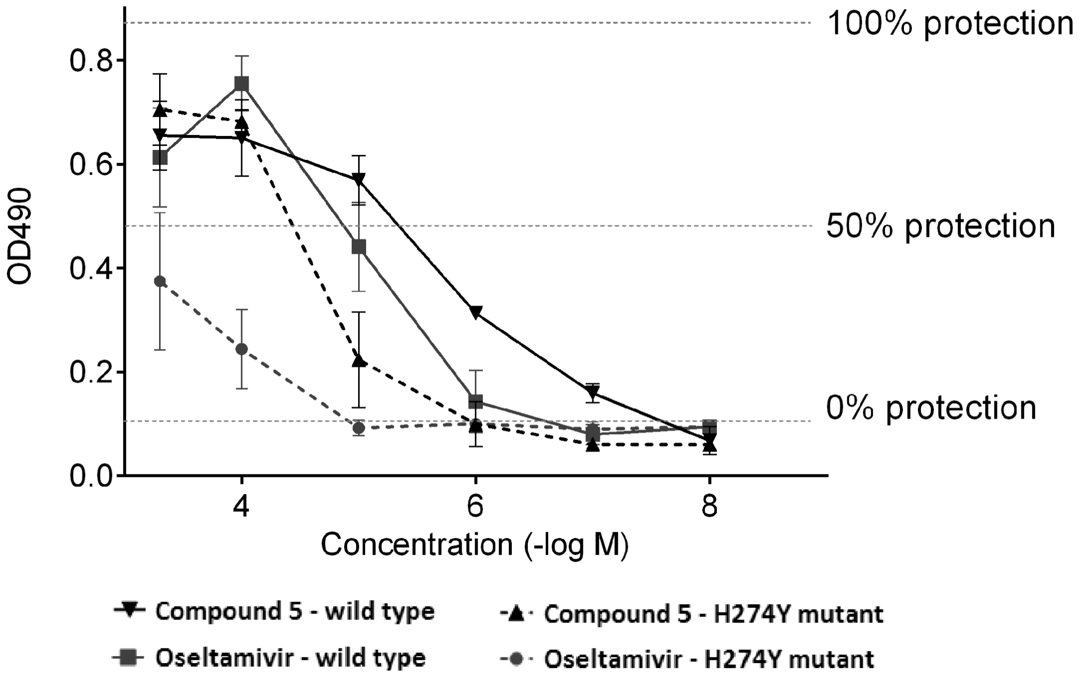

Supplement: Supplementary Information [file srep02871-s1.pdf]
